# Acute Cerebral Ischemic Infarct in a 34-Year-Old Patient After an Asymptomatic COVID-19 Infection: A Case Report

**DOI:** 10.7759/cureus.21507

**Published:** 2022-01-23

**Authors:** Mohamad Zayour, Khaled Soukarieh, Rana Al Ashkar, Mohamed AlMoussawi, Rabab Nassereldine, Ramy Atat, Bassam Mansour

**Affiliations:** 1 Cardiology, University of Balamand, Beirut, LBN; 2 Internal Medicine, Lebanese University Faculty of Medicine, Beirut, LBN; 3 Pulmonary and Critical Care Medicine, Lebanese University Faculty of Medicine, Beirut, LBN; 4 Neurology, Al Zahraa Hospital University Medical Center, Beirut, LBN; 5 Neurology, Lebanese University Faculty of Medicine, Beirut, LBN; 6 Pulmonary and Critical Care Medicine, Al Zahraa Hospital University Medical Center, Beirut, LBN

**Keywords:** sars-cov-2, asymptomatic infection, case report, ischemic strokes, covid-19 complications

## Abstract

COVID-19 is an infectious disease induced by severe acute respiratory syndrome coronavirus 2 (SARS-CoV-2), an enveloped RNA coronavirus that primarily has a tropism for the respiratory tract. Respiratory tract symptoms are frequently encountered, but many complications of this disease are still under study, including cardiovascular and neurological syndromes. The latter was linked to a severe disease presentation, but there are no reports on asymptomatic disease presentations.

A thirty-four-year-old lady presented to the emergency division for acute right-sided weakness. She was previously healthy, with no history of miscarriages. She had no previous signs or symptoms of any respiratory tract infection or other symptoms suggestive of COVID-19 infection. The physical exam revealed a complete right-sided hemiparesis with no other findings. Her initial blood workup was normal. The echocardiography and a carotid duplex ultrasound were performed and did not show any abnormality. A real-time polymerase chain reaction (PCR) for COVID-19 was negative; however, serology testing including IgM and IgG were positive, suggesting a recent COVID-19 infection. Cardiovascular complications have been reported in COVID-19 patients; however, ischemic stroke in asymptomatic COVID-19 patients has not been previously reported.

Our case highlights the risk of thrombotic complications due to SARS-CoV-2 infection even in asymptomatic COVID-19 infected patients.

## Introduction

Severe acute respiratory syndrome coronavirus 2 (SARS-COV-2) is a novel virus that first emerged in Wuhan, China, responsible for the COVID-19 disease that was declared by the WHO as a global pandemic in 2020 [1]. 

COVID-19 infected patients can have a wide range of presentations from asymptomatic to severe acute respiratory distress syndrome (ARDS) and death [2]. Other symptoms, such as loss of sense of smell or taste, were less reported yet more associated with the infection. This novel virus is still under investigation, and with the increasing knowledge of its clinical presentation, studies are now focusing on the complications that follow the disease [3].

The hypercoagulability state has been widely described in COVID-19 infected patients resulting in arterial and venous thrombosis, including cerebrovascular accident (CVA) [4]. Further reports have shown that the incidence of ischemic stroke is estimated at 2.8 % in COVID-19 infected patients and increased to 5.7% in severe SARS-CoV-2 infection [5]. Furthermore, Merkler et al. showed that the incidence of ischemic stroke is 1.6 % in COVID-19 infected patients [6]. On the other hand, little has been presented in literature on the plausibility and risk of ischemic stroke in asymptomatic COVID-19 patients [6]. Hence, we present the case of a 34-year-old woman who presented to our ED for acute ischemic stroke due to a recent asymptomatic COVID-19 infection.

## Case presentation

**Figure 1:**
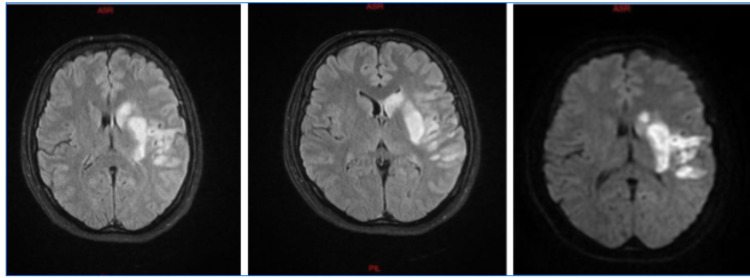
MRI results of FLAIR 1, FLAIR 2, and DWI images, respectively FLAIR - fluid-attenuated inversion recovery, DWI - diffusion-weighted image

We present a case of a 34-year-old lady without relevant past medical history who presented to the emergency division for new-onset right-side weakness that started one hour prior to her presentation. She is gravida 4, para 4 (G4P4), with no history of miscarriage or intake of oral contraceptives. Vital signs on admission were normal, and she was afebrile. Her physical neurological examination showed left side mouth deviation and right motor upper and lower limb weakness of 1/5, while the motor power was 5/5 on the left side, along with Broca's aphasia. Otherwise, her lung auscultation was completely normal. A non-contrast CT brain scan done urgently in the ER showed no abnormality. However, a brain MRI showed hyperintense signal diffusion in the left frontotemporal area in each part of the Sylvian fissure and involved the superficial and deep left Sylvian artery (Figure 1). Our patient received a recombinant tissue plasminogen activator that improved the patient transiently.

Initial labs are presented in Table 1 and showed no abnormalities. In addition to the previous, electrolytes and liver function levels were all within the normal range. She was admitted to the intensive care unit, and antiplatelet therapy (aspirin 100 mg) was started after 24 hours. Hypercoagulable state workup (Table 2) was ordered and turned out to be negative.

**Table 1:** Initial lab results WBCs - white blood cells, CRP - C-reactive protein, ESR - erythrocyte sedimentation rate, PTT - partial thromboplastin time, INR - international normalised ratio, LDL - low density lipoprotein, TSH - thyroid stimulating hormone

Lab test	Value	Comments
Hemoglobin	11.7 g/dL	
Platelets	266 000/Mcl	
WBCs	6 360/Mcl	with normal differential
CRP	0.5	
ESR	42	
PTT	24.4	
INR	0.9	
LDL	130 mg/dL	
TSH	1.5 mU/L	normal range: 0.27-4.2

**Table 2:** Hypercoagulability state lab workup ANA - antinuclear antibodies, c-ANCA - cytoplasmic anti-neutrophil cytoplasmic antibodies, p-ANCA - perinuclear anti-neutrophil cytoplasmic antibodies

Hyper-coagulability state workup
ANA, c-ANCA, p-ANCA
Anti-thrombinIII, factor II
Factor V Leiden
Lupus anticoagulant
Anticardiolipin IgG and IgM
Anti b2 glycoprotein
Homocysteine
Protein S, protein C levels

Reverse transcription polymerase chain reaction (RT-PCR) for COVID-19 was negative; however, serology for COVID -19 was positive (Table 3), knowing that the patient denied the presence of respiratory symptoms or gastrointestinal disturbances. She also denied a history of previous miscarriage or thromboembolic events. 

**Table 3:** SARS-CoV-2 serology results

Test	Results
SARS-CoV-2 IgG	9.8 AU/ml (reactive >1)
SARS-CoV-2 IgM	3.1 AU/ml (reactive >1)
Anti-SARS-CoV-2 spike protein	244.80 U/mL

We completed our workup with an MRA that confirmed the presence of acute ischemic stroke with significant compression of the left lateral ventricle. Doppler echocardiography was normal without intracardiac thrombus, with no left atrial thrombus or any direct or indirect signs of shunt presence (not confirmed by a bubble study due to financial reasons). Doppler echocardiography was normal and ruled out intracardiac shunt or thrombus of the left cavities, while duplex carotid ultrasound ruled out the presence of carotid plaques. During her hospital stay, the patient developed a tonic-clonic seizure and was treated with antiepileptic medication.

Our patient was discharged with an anticoagulant and statin, and rehabilitation was planned.

## Discussion

We presented the case of a 34-year-old woman with confirmed ischemic stroke and a recent asymptomatic COVID-19 infection.

The occurrence of thromboembolic events has been previously reported in COVID-19 [7]. Further reports suggested that the hypercoagulability is due to a severe inflammatory response mediated by a cytokine storm [8, 9]. 

A recent cohort study showed that 2 to 6% of COVID-19 infected patients develop a cerebrovascular accident [10]. Furthermore, Mao et al. reported that the incidence of ischemic stroke was estimated at 5.7 % in severe COVID-19 infection, while it was estimated at 0.8 % in those with mild disease [11]. Teuwen et al. studied the effect of the novel coronavirus infection on the coagulation cascade [12]. The cytokine storm elevates the levels of both IL-6 and CRP. These increased levels were related to an expanded risk of both stroke and thrombotic impact. 

On the other hand, the presence of an angiotensin-converting enzyme 2 (ACE2) receptor on the vascular endothelium of the cerebellum results in viral-induced vasculitis and ischemic stroke [13]. Second, the viral infection causes ACE2 receptor hindrance, therefore lowering ACE2 capacity, which diminishes the arrangement of angiotensin, weakens cerebral vascular autoregulation, and it can raise a disturbance in the vascular stream [8]. Also, the hypoxemia that results from severe COVID-19 infection may result in hypoxic brain injury.

It is well known that cardiac embolism is the most common cause of ischemic stroke in young adults [14, 15]. We presumed that the ischemic stroke in our patient is due to a COVID-19 infection after having ruled out the presence of hypercoagulability state, intracardiac shunt, and cardiac embolism. Our patient had normal vital signs on admission, and the Holter EKG also showed regular sinus rhythm, which has excluded the possibility of cardiac embolism [15].

## Conclusions

Our case is the first to report the occurrence of ischemic cerebrovascular accidents in asymptomatic COVID-19 infected patients, and it suggests that cerebral endothelial vasculitis may occur even in an asymptomatic COVID-19 infected patient.

## References

[1] World Health Organization. (2020 (2020). WHO novel coronavirus situation report 3. https://apps.who.int/iris/bitstream/handle/10665/330762/nCoVsitrep23Jan2020-eng.pdf?sequence=1&isAllowed=y.

[2] Dhama K, Khan S, Tiwari R (2020). Coronavirus disease 2019 - COVID-19. Clin Microbiol Rev.

[3] Chilamakuri R, Agarwal S (2021). COVID-19: characteristics and therapeutics. Cells.

[4] Abou-Ismail MY, Diamond A, Kapoor S, Arafah Y, Nayak L (2020). The hypercoagulable state in COVID-19: Incidence, pathophysiology, and management. Thromb Res.

[5] Trejo-Gabriel-Galán JM (2020). Stroke as a complication and prognostic factor of COVID-19. Neurología (English Edition).

[6] Merkler AE, Parikh NS, Mir S (2020). Risk of ischemic stroke in patients with coronavirus disease 2019 (COVID-19) vs patients with influenza. JAMA Neurol.

[7] Mao L, Wang M, Chen S (2020). Neurological manifestations of hospitalized patients with COVID-19 in Wuhan, China: a retrospective case series study [PREPRINT].

[8] Hess DC, Eldahshan W, Rutkowski E (2020). COVID-19-related stroke. Transl Stroke Res.

[9] Oxley TJ, Mocco J, Majidi S (2020). Large-vessel stroke as a presenting feature of Covid-19 in the young. N Engl J Med.

[10] Ellul MA, Benjamin L, Singh B (2020). Neurological associations of COVID-19. Lancet Neurol.

[11] Mao L, Jin H, Wang M (2020). Neurologic manifestations of hospitalized patients with coronavirus disease 2019 in Wuhan, China. JAMA Neurol.

[12] Teuwen LA, Geldhof V, Pasut A, Carmeliet P (2020). COVID-19: the vasculature unleashed. Nat Rev Immunol.

[13] Berger JR (2020). COVID-19 and the nervous system. J Neurovirol.

[14] Singhal AB, Biller J, Elkind MS (2013). Recognition and management of stroke in young adults and adolescents. Neurology.

[15] Moores M, Yogendrakumar V, Bereznyakova O (2020). Normal systolic blood pressure at presentation with acute ischemic stroke predicts cardioembolic etiology. J Am Heart Assoc.

